# Steerable versus nonsteerable sheath technology in atrial fibrillation ablation: A systematic review and meta‐analysis

**DOI:** 10.1002/joa3.12742

**Published:** 2022-06-03

**Authors:** Mohammed Mhanna, Azizullah Beran, Ahmad Al‐Abdouh, Omar Sajdeya, Mahmoud Barbarawi, Mahmoud Alsaiqali, Ahmad Jabri, Ahmad Al‐Aaraj, Abdulmajeed Alharbi, Paul Chacko

**Affiliations:** ^1^ Department of Internal Medicine University of Toledo Toledo Ohio USA; ^2^ Department of Internal Medicine University of Kentucky Lexington Kentucky USA; ^3^ Department of Cardiovascular Medicine University of Connecticut Farmington Connecticut USA; ^4^ Department of Internal Medicine State University of New York Downstate Medical Center Brooklyn New York USA; ^5^ Department of Cardiology Case Western Reserve University/MetroHealth Medical Center Cleveland Ohio USA; ^6^ Department of Cardiology James Cook University Hospital Middlesbrough UK; ^7^ Department of Cardiovascular Medicine University of Toledo Toledo Ohio USA

**Keywords:** atrial fibrillation, catheter ablation, pulmonary vein isolation, steerable sheath

## Abstract

**Introduction:**

Catheter placement and stability are well‐known challenges in atrial fibrillation (AF) ablation. As a result, steerable sheaths (SS) were developed to improve catheter stabilization and maintain proper catheter–tissue contact. The purpose of this systematic review and meta‐analysis is to see if employing a SS influences procedure outcome.

**Method:**

We performed a comprehensive literature search for studies that evaluated the efficacy and safety of SS compared to nonsteerable sheaths (NSS) in AF ablation. The primary outcome was the rate of atrial arrhythmia (AA) freedom by the time of the last follow‐up. The secondary outcomes were the procedure‐related complications and procedural characteristics. Risk ratio (RR) or the mean difference (MD) and corresponding 95% confidence intervals (CIs) were calculated using the random‐effects model.

**Results:**

A total of 10 studies, including 967 AF patients (mean age: 59.2 ± 11.1 years, 516 patients managed with SS vs. 454 with NSS), were included. SS group showed a higher rate of freedom of AA compared to NSS (RR: 1.19; 95% CI 1.09–1.29; *p* < .001). Both techniques had similar rate for procedural‐related complication (RR: 1.09, 95% CI 0.50–2.39; *p* = .83). The SS strategy had a shorter procedure time (MD −10.6 [min], 95% CI −20.97, −0.20; *p* = .05) but comparable fluoroscopic and radiofrequency application times to the NSS group.

**Conclusions:**

The SS for AF catheter ablation not only reduced the total procedure time but also significantly increased the rate of successful ablation while maintaining a similar safety profile when compared to the traditional NSS.

## Graphical abstract

This meta‐analysis reveals that the use of a steerable sheath for atrial fibrillation ablation resulted in a better success rate and a lower risk of late atrial arrhythmia recurrence. However, this should be weighed against the possibility of an increase in periprocedural complications and procedure costs.

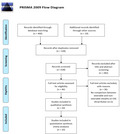

## 1 INTRODUCTION

Atrial fibrillation (AF) is the most common arrhythmia and continues to be a global health problem.^1^ In the United States, the prevalence of AF is increasing, with 12.1 million people estimated to have AF by 2030.^1^ Catheter ablation (CA) is the most common interventional treatment for AF, and it focuses on isolating the pulmonary veins (PVs).^2^ A year following ablation, at least half of the patients experience AF recurrence, likely because of electrical reconnections of isolated PVs.^2^ As a result, long‐term PV isolation (PVI) is thought to be critical for improving AF ablation clinical outcomes.

Full‐thickness scar formation represents the main challenge to maintaining long‐lasting isolation. Several factors influence the amount of energy delivered to secure effective PVs isolation, including catheter tip size, force and duration of radiofrequency ablation, irrigation mechanism, contact force (CF), and catheter stability.^3^ The degree of tissue contact of CA is a crucial component in forming a deeper and large area of scarring, where various catheter techniques have been developed to enhance procedural success.^4^

Compared to a non‐steerable sheath (NSS), a steerable sheath (SS) technology allows for more control over catheter manipulation and could theoretically provide a wider range of catheter orientations and better stability, potentially resulting in better tissue contact and thus more effective ablation lesions.^5^ However, studies analyzing the clinical outcomes of a SS technology are currently limited by small sample sizes. Therefore, we conducted this meta‐analysis to evaluate all the available evidence to better assess the efficacy and safety of the AF CA using SS compared to the traditional NSS.

## 2 METHODS

We conducted this systematic review and meta‐analysis based on the guidelines of the Preferred Reporting Items for Systematic Reviews and Meta‐analysis^6^ and Meta‐analysis of Observational Studies in Epidemiology.^7^

### 2.1 Data sources and search strategy

We performed a comprehensive search for published studies indexed in PubMed/MEDLINE, EMBASE, and Cochrane databases from inception to March 18, 2022. We also performed a manual search for additional relevant studies using references of the included articles. The following search terms were used: (“atrial fibrillation” or “AF” or “atrial arrhythmia”), (“endocardial ablation” or “catheter ablation” or “radiofrequency ablation” or “pulmonary vein isolation” or “mitral isthmus ablation”), and (“Steerable sheath” or “Robotic sheath” or “Sheath technology”). The search was not limited by language, study design, or country of origin. Table S1 describes the full search term used in each database searched.

### 2.2 Eligibility criteria

We included studies that met the following eligibility criteria: (1) peer‐reviewed cohort studies, case–control studies, or randomized controlled trials (RCTs), (2) that performed a direct comparison between SS and NSS, (3) when used for patients with AF, and (4) reported the outcomes of interest. Outcomes of interest included arrhythmia freedom at 6 months or longer, procedural related complications, or procedural characteristics. We excluded conference abstracts, single‐arm studies, case reports, and case series. Two investigators (M.M. and A.B.) independently screened and selected the studies for the final review. Discrepancies were resolved by a third investigator (A.A.).

### 2.3 Data extraction

Extracted data included study design, country and year of the study, follow‐up duration, sample size, efficacy endpoints (the freedom of atrial arrhythmia by the time of the last follow‐up), and safety endpoints (including periprocedural complications such as pericardial effusion, atrio‐esophageal fistula, cerebrovascular accident, access site complications, and death). Also, we extracted data for the number of patients who managed with SS or NSS, their age, gender, duration of AF, and baseline comorbidities (including diabetes mellitus, hypertension, coronary artery disease, body mass index) and pre‐procedural characteristics (including left ventricular ejection fraction [LVEF], left atrial [LA] diameter, percentage of paroxysmal AF, previous ablations, and CHA_2_DS_2_‐VASc score). Finally, we extracted ablation procedure details, procedural, fluoroscopic, and RF application times, as well as the postprocedural antiarrhythmic drugs (AADs) if used. Two investigators (M.M. and A.B.) independently extracted the data from the included studies. Microsoft Excel was used for data extraction. Any discrepancies were resolved by consensus.

### 2.4 Outcomes

The primary outcome of our meta‐analysis was freedom of atrial arrhythmia (AA) by the time of the last follow‐up. Total AA is defined as a composite of AF, sustained atrial tachycardia (AT), and atypical atrial flutter after the index procedure.

Our secondary outcomes included the rate of periprocedural complications through 30 days of the index procedure. We also included the following procedural characteristics in our secondary outcomes: total procedure time, fluoroscopy time, and RF application time.

### 2.5 Statistical analysis

We performed a meta‐analysis of the included studies using Review Manager 5.3 (Cochrane Collaboration, Copenhagen, The Nordic Cochrane Centre) and Comprehensive Meta‐Analysis (Biostat). The median and interquartile range were converted to mean and SD where applicable.^8^ The random‐effects model was used to calculate the pooled risk ratio (RR) and mean difference (MD) with the corresponding confidence intervals (CI) for proportional and continuous variables, respectively. A value of *p* < .05 was considered statistically significant. The heterogeneity was evaluated using the *I* ^2^ statistic as defined by the Cochrane handbook for systematic reviews. *I* ^2^ value of ≥50% was considered significant heterogeneity for all outcomes.^9^

### 2.6 Sensitivity and subgroup analyses

We performed subgroup analyses of the primary outcome based on the usage of AADs after the blanking period, the strategy of SS delivery (robotic or manual), ablation status (first time or repeated ablation), or the use of the CF sensing catheters if at least two studies reported the outcome. We also performed subgroup analysis based on the study design (RCTs vs. observational studies). To confirm the robustness of our results, sensitivity analysis for AA freedom and procedure‐related complications using leave‐one‐out meta‐analysis was performed to see if it had a significant influence on the meta‐analysis result (i.e., jack‐knife sensitivity analysis).

### 2.7 Quality and bias assessment

The Jadad composite scale was used to assess the methodological quality of the clinical trials based on randomization, blinding, and withdrawals.^10^ The scale ranged from 0 to 5 points.^10^ Studies with a total score of ≥3 were considered to have a low risk of bias. The Newcastle–Ottawa Quality Assessment Scale was used to assess the quality of the observational studies based on the selection of the study groups, comparability of study groups, and ascertainment of exposure/outcome.^11^ Studies with total scores of ≥6 were considered to have a low risk of bias. For outcomes reported by ≥8 studies, publication bias was assessed qualitatively by visual inspection of the funnel plot and quantitatively by Egger's regression analysis. Two authors (A.B. and M.M.) independently assessed each study for bias. Discrepancies were resolved by a third reviewer (O.S.).

## 3 RESULTS

### 3.1 Study selection

**FIGURE 1:**
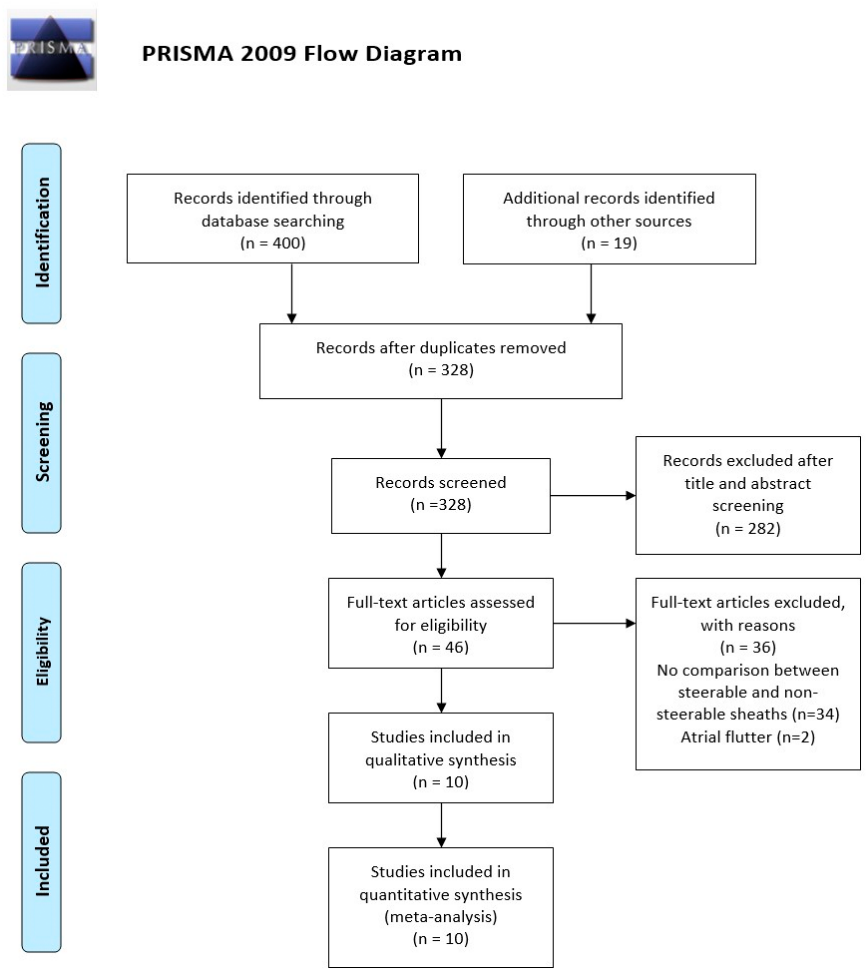
PRISMA flow diagram for the selection of studies.

A total of 419 studies were retrieved by our search strategy. Among these, 46 were eligible for the systematic review. Subsequently, we excluded 36 studies because of single‐arm studies reporting either SS or NSS only or being conducted only in atrial flutter patients. Finally, 10 studies met our inclusion criteria and were included in the meta‐analysis.^12^, ^13^, ^14^, ^15^, ^16^, ^17^, ^18^, ^19^, ^20^, ^21^ Figure 1 shows the PRISMA flow chart that illustrates how the final studies were selected.

### 3.2 Study characteristics

Table 1 shows the characteristics of the 10 studies that were included in our meta‐analysis. The studies included a total of 970 AF patients, of whom 516 patients managed with SS versus 454 with NSS. All the included studies were published between July 2008 and February 2022 and only included patients with AF. Based on the country of origin, two studies originated from the United Kingdom, two from Japan, two from China, one from Germany, one from Monaco, one from Canada, and one from Switzerland. Based on study design, three studies were RCTs,^17^, ^19^, ^20^ three were retrospective cohorts,^12^, ^14^, ^21^ two were prospective cohorts^13^, ^16^ and two were case–control studies.^15^, ^18^ The mean age was 59.2 ± 11.1 years, and males represented 62.7% of total patients. The mean AF history was 47.5 ± 52.5 months. Around 61.1% of the entire study population had paroxysmal AF. Table 2 summarizes the baseline comorbidities, including LVEF, LA diameter, and CHA_2_DS_2_‐VASc score, and shows no statistically significant differences between the two groups regarding their baseline characteristics.

**TABLE 1:** Characteristics of studies included in the meta‐analysis

Study	Design	Origin	Total patients (SS/NSS), *n*	Follow‐up duration, months	Inclusion criteria	AAD allowed after blanking period^a^	AA detection	SS used	NSS used
Deyell, 2020	RC	Multicentric Canada	(52, 33) 85	12	PVI alone (no adjunctive linear or complex fractionated electrogram ablation)	No	24 h Holter monitoring q3 months	Agilis™ NxT small or medium curve sheaths^b^	8.5 French SL0 or SL1 sheaths^c^
Errahmouni, 2015	PC	Single center Monaco	(45, 37) 82	>9	Consecutive patients who underwent AF ablation using MN coupled with the NSS group were prospectively included	Yes	24 h Holter monitoring q3 months for the first year, the q6 months afterward	Robotic deflectable sheath	Fast‐Cath SL1^c^
Guo, 2021	RC	Single center China	(53, 67) 120	6	Paroxysmal AF First‐time ablation	No	(ECG) or 24‐h Holter monitoring	Vizigo sheath^d^	Swartz sheath^e^
Luo, 2022	Case–control	Single center China	(55, 55) 110	1 day	AF refractory to at least one antiarrhythmic	NR	NR	MobiCath^f^	Fast‐Cath SR0^e^
Masuda, 2016	PC	Single center Japan	(57, 33) 90	12	Patients who underwent an initial ablation for AF	No	24 h Holter monitoring q3 months	Agilis™ NxT sheaths^b^	Swartz sheath^e^
Matsuo, 2011	RCT	Single center Japan	(40, 40) 80	12	Persistent AF	Yes	(ECG) or 24‐h Holter monitoring q3 months Event recorder (available for 5 days) if symptomatic without AA documentation	Agilis™ NxT sheaths^b^	8 French SL0 sheaths^c^
Piorkowski, 2008	Case–control	Switzerland	(83, 83) 166	6	Cases where matched with previously identified controls who treated with nonsteerable sheaths	Yes	7‐day Holter ECG at 3 and 6 months	Agilis™ NxT sheaths^b^	Mullins^g^
Piorkowski, 2011	RCT	2 centers Germany	(63, 60) 123	6	(1) Paroxysmal or persistent symptomatic AF (documented on ECG) (2) Refractory to at least 1 AAD (3) LA diameter <60 mm	Yes	7‐day Holter ECG at 3 and 6 months	Agilis™ NxT sheaths^b^	SL0 sheaths^c^
Rajappan, 2009	RCT	Single center United Kingdom	(27) 54	6	(1) Paroxysmal or persistent symptomatic AF (documented on ECG) (2) Refractory to at least 1 AAD (3) First time ablation	No	7‐day Holter monitor	Agilis™ NxT sheaths^b^	Mullins^g^
Ullah, 2015	RC	Single center United Kingdom	(41, 19) 60	12	Persistent AF, First time ablation	No	NR	Agilis™ NxT sheaths^b^ or Artisan Extend Control Catheter^h^	Mullins^g^

Abbreviations: AA, atrial arrhythmia; AAD, antiarrhythmic drug; AF, atrial fibrillation; LA, left atrium; MN, remote magnetic navigation; NR, not reported; NSS, nonsteerable sheath; PC, prospective cohort; PVI, pulmonary vein isolation; RC, retrospective cohort; RCT, randomized controlled trial; SS, steerable sheath.

^a^ Unless AA recurrence observed.

^b^ Abbott Medical.

^c^ Abbott Medical, St. Paul, MN, USA.

^d^ Biosense Webster Inc., Irvine, CA, USA.

^e^ St. Jude Inc., St. Paul, MN, USA.

^f^ Biosense Webster Inc.

^g^ Cook Inc., Bloomington, IN, USA.

^h^ Hansen Medical Inc.

**TABLE 2:** Baseline patients characteristics included in the meta‐analysis

	No of studies	All patients (*N* = 970)	Steerable sheath (*N* = 516)	Nonsteerable sheath (*N* = 454)	*p*‐value
Age, year	10	60.7 ± 9.8	59.2 ± 11.1	59.5 ± 11.1	NS (.42)
Male	10	62.7% (608/970)	63.4% (327/516)	61.9% (281/454)	NS (.64)
BMI	4	25 ± 3.8	25 ± 3.8	25 ± 3.7	NS (.85)
Hypertension	7	46% (338/734)	45.4% (179/394)	46.8% (159/340)	NS (.72)
Diabetes mellitus	4	10.4% (39/374)	11.8% (24/203)	8.8% (15/171)	NS (.34)
CAD	4	11.3% (53/469)	12.1% (29/240)	10.5% (24/229)	NS (.58)
CHADS2 score	4	1.84 ± 1.4	1.81 ± 1.4	1.87 ± 1.5	NS (.72)
LA diameter, mm	10	40.6 ± 7.7	40.5 ± 8.2	40.7 ± 7.1	NS (.77)
LVEF, %	7	63.5 ± 7.8	63.4 ± 8	63.6 ± 7.7	NS (.79)
Paroxysmal AF, %	10	61.6% (598/970)	59.3% (306/516)	64.3% (292/454)	NS (.11)
AF history, m	5	47.5 ± 52.5	46.7 ± 54.6	48.2 ± 50.1	NS (.75)
Procedure time, min	7	175.7 ± 93.4	172.6 ± 62.9	179 ± 117	.05
Fluoroscopy time, min	7	21.3 ± 19.8	19.8 ± 17	22.8 ± 22.2	NS (.27)
RF application time, min	5	41.7 ± 14.3	40.7 ± 14	42.8 ± 14.6	NS (.12)

Abbreviations: AF, atrial fibrillation; BMI, body mass index; CAD, coronary artery disease; LA, left atrium; LVEF, left ventricular ejection fraction; NS, not significant; RF, radiofrequency.

In terms of the ablation procedure, the majority of the studies reported at least 3 weeks of uninterrupted anticoagulation therapy with warfarin (target international normalized ratio, 2–3) or direct oral anticoagulants before the procedure. Transesophageal echocardiography was performed before the procedure to rule out left atrium thrombus. Mapping and ablation were performed under the guidance of 3D mapping systems (the CARTO™ mapping system “Biosense Webster, Diamond Bar, CA”) in most cases. In either a unipolar or bipolar mode, radiofrequency alternating current was provided. In the majority of the investigations, open irrigation tip catheters were used. In terms of lesion set ablation, all the studies included performed PVI. If AF persisted following PVI, ablation of fractionated electrograms and application of complete lines were done at the discretion of the physician in some cases. The standard ablation setting consisted of an upper‐temperature limit of 42–50°C, a power of 25–40 W, and a flow rate of 17–30 ml/min. Near to the esophagus, power delivery was reduced. Individual ablation features for each included study are shown in Table S2.

All studies defined AF recurrence as any atrial arrhythmia lasting more than 30 seconds after 13‐ to 3‐month blanking period. The average follow‐up duration was 6 months (range 6–12 months). The assessment of atrial recurrence was made mainly through Holter monitoring ranging from 24 h to 7 days. None of the included studies reported utilizing implantable loop recorder monitoring or pacemaker device interrogation.

### 3.3 Outcomes of interest

#### 3.3.1 Atrial arrhythmia freedom

**FIGURE 2:**
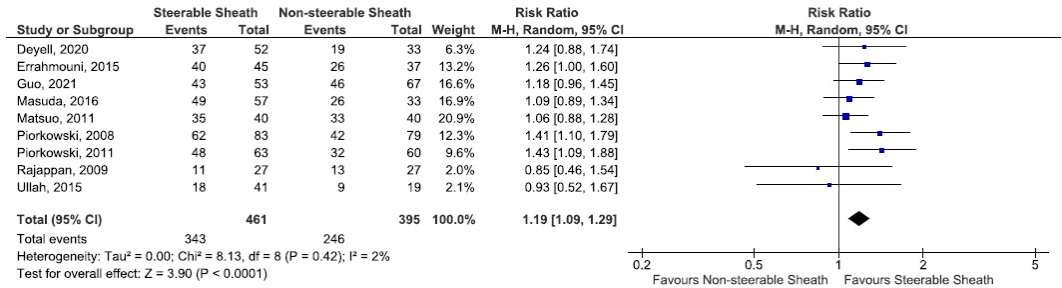
Forest plot comparing steerable sheath and nonsteerable sheath regarding the freedom of atrial arrhythmia by the time of last follow‐up.

Across the nine studies^12^, ^13^, ^14^, ^16^, ^17^, ^18^, ^19^, ^20^, ^21^ that reported the rate of freedom of atrial arrhythmia by the time of last follow‐up (average of 6 months), 74.4% (343/461) of patients managed with SS attained AA freedom compared to 62.3% (246/395) in the NSS group. This difference was statistically significant favoring the SS technique (RR: 1.19; 95% CI 1.09–1.29; *p* < .001, *I* ^2^ = 2%, Figure 2).

The results on the subgroup analysis of RCTs^17^, ^19^, ^20^ showed that the proportion of patients who achieved AA freedom was higher in the SS group but did not reach statistical significance (RR: 1.15; 95% CI 0.89–1.48; *p* = .30). However, the test of the subgroup difference was not significant, indicating that the SS technique was more successful than the NSS strategy in achieving AA freedom regardless of whether the studies were RCTs or observational studies (test of subgroup differences: *χ* ^2^ = 0.13, degree of freedom = 1, *p* = .72) (Figure S1).

We also performed a subgroup analysis based on whether the AADs were allowed after the blanking period^13^, ^17^, ^18^, ^19^ or not, and it showed that using AADs enhanced the AA freedom success rate (RR: 1.26; 95% CI 1.08–1.47; *p* = .004). However, the test of the subgroup difference was not significant, indicating that the SS technique was more successful than the NSS strategy in achieving AA freedom regardless of whether the AADs were allowed or not (test of subgroup differences: *χ* ^2^ = 1.23, degree of freedom = 1, *p* = .27) (Figure S2).

Four of the included studies^14^, ^16^, ^20^, ^21^ reported SS utilization in patients who underwent first‐time ablation, results showed that patients managed with the SS had a higher rate of AA freedom compared to the NSS group, although this difference did not reach a statistical significance (RR: 1.11; 95% CI 0.96–1.27; *p* = .16). However, the test of the subgroup difference was not significant, indicating that the SS technique was more successful than the NSS strategy in achieving AA freedom regardless of whether the CA was used for the first time or was a repeated one (test of subgroup differences: *χ* ^2^ = 1.63, degree of freedom = 1, *p* = .20) (Figure S3).

Another subgroup analysis looked at whether CF sensing catheters were utilized during the ablation procedure^12^, ^14^, ^16^, ^21^; It found that using CF catheters did not enhance the AA freedom rate (RR: 1.14; 95% CI 1.00–1.30; *p* = .05). Regardless of whether CF sensing catheters were used or not, the SS technique was found to be superior to the NSS strategy (test of subgroup differences: *χ* ^2^ = 0.59, degree of freedom = 1, *p* = .44) (Figure S4).

Finally, two of the included studies^13^, ^21^ reported utilization of robotic assistant SS technology and showed that using robotic technology further enhanced the AA freedom success rate when compared to manually guided SS (RR: 1.24; 95% CI 1.00–1.55; *p* = .05). However, regardless of whether SS was manually or robotic guided, they maintained their efficacy over the NSS (test of subgroup differences: *χ* ^2^ = 0.19, degree of freedom = 1, *p* = .66) (Figure S5).

#### 3.3.2 Procedure‐related complications

**FIGURE 3:**
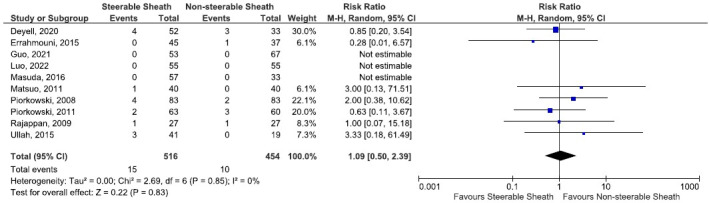
Forest plot comparing steerable sheath and nonsteerable sheath regarding the periprocedural adverse events.

There were 15 procedure‐related complications (2.9%) in the SS group, including one vasovagal reaction, two pericarditis, two minor pericardial effusions, three cardiac tamponades, one moderate PV stenosis of no clinical significance, one stroke, one venous thrombosis, and two access site pseudoaneurysm. In the NSS, 10 procedure‐related complications were reported (2.2%). The rate of periprocedural complications was similar between the two groups (RR: 1.09, 95% CI 0.50–2.39, *p* = .83, *I* ^2^ = 0%, Figure 3). Furthermore, seven access site‐related complications were observed in the SS group, including three recurrent groin delayed bleeding (not requiring intervention) and two pseudoaneurysms, compared to only two complications observed in the NSS group. However, this difference did not reach a statistical significance (RR: 1.68, 95% CI 0.46–6.18, *p* = .43) (Figure S6). The two groups did not have any procedure‐related mortality, significant PV stenosis, phrenic nerve palsy, or esophageal fistula.

#### 3.3.3 Procedural characteristics

**FIGURE 4:**
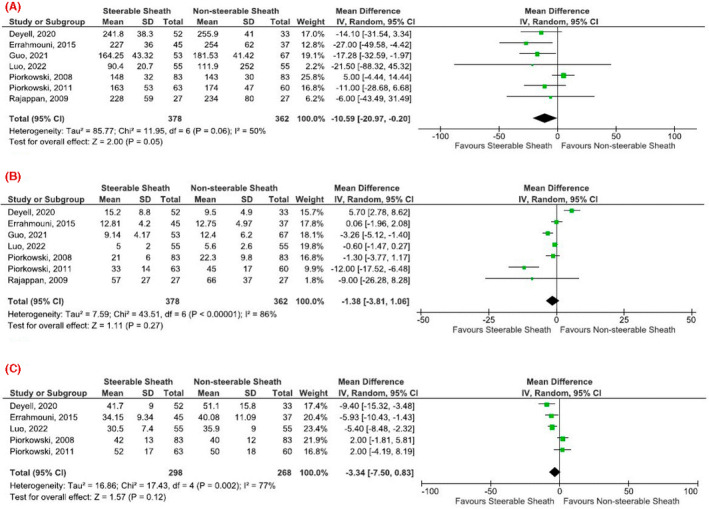
Forest plot comparing steerable sheath and nonsteerable sheath regarding (A) procedure time, (B) fluoroscopy time, and (C) radiofrequency application time.

The SS group had shorter procedure time than the NSS group (MD −10.59 min, 95% CI −20.97, −0.20, *p* = .05, *I* ^2^ = 50%, Figure 4A). The leave‐one‐out sensitivity analysis for procedure time showed that excluding the study by Piorkowski et al.^18^ resulted in *I* ^2^ = 0% with consistent results, suggesting that the study by Piorkowski et al. was mainly the reason for the heterogeneity in procedure time (Figure S7).

However, mean fluoroscopic and radiofrequency application times did not differ significantly between both treatment groups (MD −1.38 min, 95% CI −3.81, 1.06, *p* = .27, *I* ^2^ = 86%, Figure 4B) and (MD −3.34 min, 95% CI −7.5, 0.83, *p* = .12, *I* ^2^ = 77%, Figure 4C), respectively. A leave‐one‐out sensitivity analysis for both fluoroscopic and radiofrequency application times showed consistent results, as shown in Figures S8 and S9, respectively.

### 3.4 Quality and bias assessment

Quality assessment scores of the RCTs and observational studies are summarized in Table S3. All studies scored low to moderate in quality assessment. The funnel plots for AA freedom and procedural complications appeared symmetric by visual inspection (Figures S10 and S11), and Egger's regression analysis did not show evidence of publication bias (*p* = .12 and *p* = .92 for the AA freedom and procedural complications, respectively).

## 4 DISCUSSION

This study is a systematic review and meta‐analysis of all studies comparing the efficacy and safety of the SS technology to traditional NSS when used in AF CA. Our meta‐analysis found that using SS increased atrial tachyarrhythmia freedom and was associated with a shorter overall procedure duration while having a similar safety profile to the NSS strategy. In both strategies, the fluoroscopic and RF application times were comparable.

The recurrence rate of AF after CA varies greatly between studies; early recurrences (within the first 3 months) occur in about half of individuals after CA.^22^ In the CIRCA‐DOSE trial, late recurrence (beyond 3 months) was seen in more than 40% of patients, as determined by continuous rhythm monitoring.^2^ Thus, repeated procedures and the use of maintenance antiarrhythmic medications are usually necessary to achieve satisfactory success rates.^23^ The rate of repeated ablation procedures may reach up to 80%.^24^ Despite added strategies to attain durable PVI, the success rate did not remarkably improve.^25^

The ability to establish durable transmural lesion sets is a major limiting factor in CA's success. The contact between the ablation catheter and the atrial myocardium is one of the key determinants of efficient lesion formation during radiofrequency (RF) ablation.^26^ Several techniques have been developed to improve tissue contact during RF ablation. To improve the maneuverability and stability of the ablation catheter, one of the most common ways is to use a steerable guiding sheath.^27^ Catheter movement is a common problem that complicates both complex and basic ablation and mapping procedures.^28^ As a result, efforts and advancements have centered on catheter stabilization strategies to reduce harmful motion and improve catheter–tissue contact reliability. The SS has aided in the stabilization of the ablation catheter during AF ablation to a significant extent. These long, rigid sheaths usually allow for flexion and counterflexion in a single plane, giving the operator more control over the catheter while also offering more support and stability.^14^, ^18^ SS have been demonstrated to boost CF, enable mapping and ablation, and shorten procedure duration; hence these characteristics have translated into superior mid‐ and long‐term clinical outcomes as compared to the conventional NSS.^14^, ^20^, ^21^ However, respiratory motion is known to impact catheter stability and catheter–tissue contact, particularly in the roof of the LA. Yet, the inflexible shaft of SS may give limited flexibility, which possess a major disadvantage of SS when used on the LA roof.^28^

In our study, the rate of late AA freedom for the SS group was significantly higher than NSS one (77.4% vs. 62.3%, respectively). Maintaining a steady contact between the tip of the catheter and the ablation points, which is strengthened by precise adjustment and strong support given by the SS, could explain the increased success of SS technology. The minimum CF of ablation points, as shown in the EFFICAS I multi‐center study, is a powerful factor in predicting the gap, as a small CF may lead to ineffective ablation.^29^ Thus, with the stability provided by SS, the effectiveness of complete pulmonary isolation is enhanced with improvement of lesion continuity and transmurality, resulting in more durable efficacy and aid in maintaining a sinus rhythm. In a single‐arm retrospective study including 679 AF patients which investigated the effectiveness of SS technology using the circumferential PVI as the ablation strategy, the success rate reached 76% at 12 months, however, about 45% and 21% of the patients received AADs for the first and sixth months.^30^ Although the baseline characteristics of our cohort of patients are quite homogenous, we pooled the success rate of the SS technology from a heterogeneous group of ablation strategies, lesion set, and proceduralist. Matsuo et al. achieved bidirectional conduction block across the mitral isthmus line more often with a SS (98% vs. 78%), but this did not affect freedom from recurrent AF (53% vs. 43%), both groups underwent a standardized additional LA ablation protocol, which included segmental PVI.^17^ Additional complex fractionated atrial electrogram ablation if required was performed in three of the included studies.^13^, ^20^, ^21^ However, most of the included studies used the circumferential PV ablation as the main ablation strategy, with no significant ablation set differences between the steerable and NSS groups, suggesting that the observed difference between the two groups may reflect true clinical significance. In addition, we examined the effect of variable parameters in our subgroup analyses including the use of CF sensing catheter, utilization of robotic assistance, the use of AADs, and the designs of the included studies, with our results remaining consistent across different subgroups.

Regarding the safety profile of the two strategies, although the periprocedural‐related adverse events were numerically higher in the steerable group, this did not reach a statistically significant level; nonetheless, because the two groups had such a small number of events, type II error cannot be ignored. Furthermore, we found two cases of pericarditis, two cases of minor pericardial effusions, and three cases of cardiac tamponade in the SS group, suggesting that the risk of cardiac tamponade when using a SS is higher because of the increased catheter to tissue contact provided by the SS and its stiff tip. Again, the occurrence rate is too low to draw any firm conclusions about the SS technology's safety. Our results on the safety profile are consistent with Hiner and Shah, who examined the utility of two commercially available SS on CF stability during PVI and found no major procedure‐related adverse effects.^31^ Furthermore, no procedure‐related complications were found in an RCT that comprised patients with typical atrial flutter and nine patients with persistent AF who received cavotricuspid isthmus ablation.^32^ Other RF ablation techniques, such as high power and short duration (HPSD), are also available, in which a higher CF can cause steam pop. As a result, the safety of SS in HPSD ablation may be questionable.^33^

In our study, the SS group had a statistically significant advantage in procedure time, but not in fluoroscopic or RF application times. However, considering the heterogeneity of procedure time recording and definitions across the included studies, the magnitude of such a difference (10–15 min) may lack clinical importance. In the Matsuo et al. study, although the total RF duration and the total amount of RF energy to establish a bidirectional block on the CTI was significantly shorter and smaller in the SS group when compared to the NSS, the total procedure time was comparable between the two groups.^32^ Finally, the financial constraints could not be overlooked, considering the large cost associated with the usage of SS.

There are certain limitations to our meta‐analysis. First, the ablation procedures were not standardized among the included studies; however, in most of the study cohort, the CVPA was the main ablation strategy. Second, in most of the studies, only symptomatic recurrences proven by EKG or Holter monitor were counted as failures; no studies employed an implantable loop recorder to confirm the AA recurrence. Third, the included trials were of a single‐blinded design. Therefore, investigator bias cannot be undermined. Fourth, because of the insufficient data and differences in the definitions presented by the included studies, we were unable to analyze the impact of SS technology on CF parameters. Nevertheless, two studies^14^, ^21^ clearly stated the mean CF, both demonstrating that the NSS group's mean total CF was significantly lower than the SS group. Guo et al reported a CF range of 10–30 g, which was significantly greater in the SS group.^14^ Deyell et al. also looked at catheter stability with less lesions having >10% of the time with a force 10 g and found that the SS group performed considerably better (overall odds ratio [OR] for SS vs. NSS: 0.56, 95% CI: 0.35–0.89, *p* = .01).^12^ Lastly, we could not assess the cost‐effectiveness of the SS strategy given the limited data provided by the included studies.

However, there are several strengths to our meta‐analysis. First, to the best of our knowledge, this is the first meta‐analysis to systematically investigate the utility of SS technology in AF ablation. Second, we performed a subgroup analysis for the late atrial arrhythmia recurrence rate based on variable parameters in our subgroup analyses, including the use of CF sensing catheter, utilization of robotic assistance, the use of AADs, and the designs of the included studies, with our results remained consistent across different subgroups. In addition, low heterogeneity was found in the measurement of our clinical and safety outcomes.

In conclusion, our meta‐analysis demonstrated that using a SS for AF ablation resulted in a better success rate and a lower risk of late atrial arrhythmia recurrence. However, this should be weighed against the possibility of an increase in periprocedural complications and procedure costs. Prospective large‐scale randomized trials are needed to validate these results.

## AUTHOR CONTRIBUTIONS

Mohammed Mhanna and Azizullah Beran conceived and designed the study, drafted the manuscript and critically revised the manuscript. Mohammed Mhanna, Paul Chacko, Mahmoud Barbarawi, Ahmad Al Aaraj, and Ahmad Al‐Abdouh designed the study, collected, analyzed, and interpreted the data. Abdulmajeed Alharbi, Mahmoud Alsaiqali, Ahmad Jabri, and Omar Sajdeya collected the data and reviewed the literature. All authors read and approved the final manuscript.

## CONFLICT OF INTEREST

The authors declared no conflict of interest.

## IRB APPROVAL

This study was deemed exempt by the Institutional Review Board of the University of Toledo, as it was a meta‐analysis of published studies that included de‐identified patient information.

## Supporting information


Appendix S1
