# Supplementary material for: Steerable versus nonsteerable sheath technology in atrial fibrillation ablation: A systematic review and meta‐analysis
Source: J Arrhythm. 2022 Jun 3;38(4):570–9. doi: 10.1002/joa3.12742 (PMC9347204; doi:10.1002/joa3.12742)
Supplement: Supplementary file 1 — Appendix S1 [file JOA3-38-570-s001.docx]

# **Supplementary Appendix**

**Steerable Versus Non-Steerable Sheath Technology in Atrial Fibrillation Ablation: A Systematic Review and Meta-Analysis**

**Supplementary tables**

| Title | Page |
| --- | --- |
| Supplementary Table 1: Search strategy used in each database searched. | 3 |
| Supplementary Table 2: Ablation procedure characteristics of the included studies in the meta-analysis. | 4-5 |
| Supplementary Table 3: Quality assessment of the included studies in the meta-analysis. | 6 |

**Supplementary figures:**

| Title | Page |
| --- | --- |
| Supplementary Figure 1: Subgroup analysis of comparing the steerable and non-steerable sheaths based on the study design (Randomized control trials vs. observational studies) | 7 |
| Supplementary Figure 2: Subgroup analysis of comparing the steerable and non-steerable sheaths based on whether anti-arrhythmic drugs were allowed or not after the blanking period. | 8 |
| Supplementary Figure 3: Subgroup analysis of comparing the steerable and non-steerable sheaths based on ablation status (first time or repeated ablation) | 9 |
| Supplementary Figure 4: Subgroup analysis of comparing the steerable and non-steerable sheaths based on the use of contact force sensing catheters. | 10 |
| Supplementary Figure 5: Subgroup analysis of comparing the steerable and non-steerable sheaths based on the steerable sheath’s guidance (robotic assisted vs. manually guided) | 11 |
| Supplementary Figure 6: Forrest blot showing the access-site related complications between the steerable and non-steerable sheath groups. | 12 |
| Supplementary Figure 7: A sensitivity analysis of the procedure time outcome, which showed no heterogeneity after removing the Poprkowski (2008) study. | 13 |
| Supplementary Figure 8: Leave-one-out sensitivity analysis for the fluoroscopic time. | 14 |
| Supplementary Figure 9: Leave-one-out sensitivity analysis for the radiofrequency application time. | 15 |
| Supplementary Figure 10: Funnel plots showing publication bias analysis of studies that assessed the effect of steerable sheath versus non-steerable sheath regarding atrial arrythmia freedom | 16 |
| Supplementary Figure 11: Funnel plots showing publication bias analysis of studies that assessed the effect of steerable sheath versus non-steerable sheath regarding procedural complications | 17 |

**Supplementary table 1:** Search strategy used in each database searched.

| Database | Search Strategy | Articles retrieved |
| --- | --- | --- |
| PubMed/MEDLINE | ("atrial fibrillation"[All Fields] OR "af"[All Fields] OR "atrial arrythmias"[All Fields]) AND ("catheter ablation"[All Fields] OR "endocardial ablation"[All Fields] OR "radiofrequence"[All Fields] OR "ablation"[All Fields] OR "pulmonary vein isolation"[All Fields] OR "mitral isthmus line"[All Fields]) AND ("steerable sheath"[All Fields] OR "steerable"[All Fields] OR "Robotic sheath"[All Fields] OR "Sheath technology"[All Fields]) | 77 |
| Embase | ('atrial fibrillation'/exp OR 'atrial fibrillation' OR 'heart atrium arrhythmia'/exp OR 'heart atrium arrhythmia' OR af) AND ('catheter ablation' OR 'endocardial ablation' OR 'radiofrequency ablation' OR 'pulmonary vein isolation' OR 'mitral isthmus ablation') AND ('steerable catheter' OR 'steerable sheath' OR steerable OR 'robotic sheath' OR 'sheath technology') | 301 |
| Cochrane CENTRAL | "atrial fibrillation" OR "AF" OR "atrial arrythmia" in Title Abstract Keyword AND "catheter ablation" OR "endocardial ablation" OR "radiofrequence" OR "ablation" OR "pulmonary vein isolation" OR "mitral isthmus line" in Title Abstract Keyword AND "steerable sheath" OR "steerable" OR "Robotic sheath" OR "Sheath technology" in Title Abstract Keyword | 22 |

**Supplementary table 2**: Ablation procedure characteristics of the included studies in the meta-analysis.

| Study | Ablation type | Ablation set | Ablation | Energy Source | Irrigation | PVI | Antral ablation | roof line | posterior wall | CTI line | Mitral line | CS ablation | VC isolation | CFAE | LOM | LAA | right atrium |
| --- | --- | --- | --- | --- | --- | --- | --- | --- | --- | --- | --- | --- | --- | --- | --- | --- | --- |
| Deyell, 2020 | Force-guided RF ablation  The CARTO 3™ mapping system^a^ was used in combination with a force sensing catheter^b^ | Index PV isolation with a force-sensing catheter.  Force of 10–40 g for each ablation lesion. | RFA, CF  Point by Point | NR | NR | ++ | ++ | _ | _ | _ | _ | _ | _ | _ | _ | _ | _ |
| Errahmouni, 2015 | RF ablation  AF ablation using MN^c^  The CARTO 3™ mapping system^a^ was used | CPVI was performed in all patients. Additional ablation was used for persistent AF. | RFA,  Point by Point | NR | External | ++ | ++ | + | _ | _ | _ | + | _ | + | _ | _ | + |
| Guo, 2021 | Force-guided RF ablation  The CARTO 3™ mapping system^a^ was used in combination with a force sensing catheter^b^ | The ablation index (AI) was adopted to guide the RF procedure.  Energy was with a flow limit of 20 mL/min, power limit of 40 W, and the temperature set to 50 °C. | RFA, CF | NR | NR | ++ | ++ | NR | NR | NR | NR | NR | NR | NR | NR | NR | NR |
| Luo, 2022 | RF ablation  AF ablation using MN^c^  The CARTO 3™ mapping system^a^ was used | CPVI was performed in all patients.  Additional ablation was performed if needed. | RFA | NR | Open | ++ | ++ | NR | NR | NR | NR | NR | NR | + | NR | NR | NR |
| Masuda, 2016 | Force-guided RF ablation  The CARTO 3™ mapping system^a^ was used in combination with a force sensing catheter^b^ | CPVI was performed in all patients.  Energy was transmitted through with a flow limit of 17 mL/min, power limit of 30 W, and the temperature set to 42 °C. | RFA, CF | NR | Open | ++ | ++ | NR | NR | NR | NR | NR | NR | NR | NR | NR | NR |
| Matsuo, 2011 | An open irrigated RF | MI ablation with an open irrigated catheter with a power limit of 25 W, and the temperature set to 45 °C.  Additional epicardial ablation was performed if needed.  CPVI, Linear roof, LA, and CTI ablation were performed in all patients. | RFA | Bipolar | Open | ++ | ++ | + | - | ++ | ++ | ++ | - | - | - | - | - |
| Piorkowski, 2008 | RF ablation using the CARTO 3™ mapping system^a^ | CPVI was performed in all patients.  Additional ablation was performed if needed.  Energy was with a flow limit of 30 mL/min, power limit of 40 W, and the temperature set to 50 °C. | RFA | Unipolar | Open | ++ | ++ | + | NR | NR | + | NR | NR | NR | NR | NR | NR |
| Piorkowski, 2011 | RF ablation using the CARTO™ mapping system^a^ or NavX.  Unipolar mode | CPVI was performed in all patients.  Additional ablation was performed if needed.  Energy was with a flow limit of 30 mL/min, power limit of 40 W, and the temperature set to 50 °C. | RFA | Unipolar | Open | ++ | ++ | + | + | + | + | - | - | - | - | - | - |
| Rajappan, 2009 | RF ablation using the CARTO™ mapping system^a^ | LACA with additional ablation was performed if needed. | RFA | Bipolar | Open | ++ | ++ | NR | NR | + | NR | + | NR | + | NR | NR | NR |
| Ullah, 2015 | Force-guided RF ablation  RF ablation using the CARTO™ mapping system^a^  MN was utilized in one group of the patients. | WACA was performed in all patients.  Additional ablation was performed if needed.  Power limit of 30 W, and the temperature set to 48 °C. | RFA, CF | Bipolar | NR | ++ | ++ | NR | NR | NR | NR | NR | NR | + | NR | NR | NR |

a (Biosense Webster, Diamond Bar, CA)

b (Thermocool SmartTouch™ or Thermocool SmartTouch Surround Flow™)

c Niobe ES, Stereotaxis; Stx

AADs=antiarrhythmic drugs, CF: Contact Force, CFAEs=complex fractionated atrial electrograms, CS=coronary sinus, CTI=cavotricuspid isthmus, LA=left atrium, LAA=left atrial appendage, LOM=ligament of Marshall, PVs=pulmonary veins, RFA=radiofrequency ablation, SVC=superior vena cava,

++: performed in all patients

+: Performed when needed

-: Not performed.

NR: Not reported.

Supplementary table 2: Quality assessment of the included studies in the meta-analysis.

| Cohort studies | Selection | | | | Comparability | Outcome | | | | Quality score |
| --- | --- | --- | --- | --- | --- | --- | --- | --- | --- | --- |
|  | Representativeness of the exposed cohort | Selection of non-exposed cohort | Ascertainment of exposure | Demonstration that outcome of interest was not present at start of study | Comparability of the cohorts on the basis of design or analysis | Assessment of outcome | Was follow up long enough for outcomes to occur | | Adequacy of follow up cohorts |  |
| Deyell, 2020 | 1 | 1 | 1 | 1 | 1 | 1 | 1 | | 1 | 8 |
| Errahmouni, 2015 | 1 | 1 | 1 | 1 | 1 | 1 | 0 | | 1 | 7 |
| Guo, 2021 | 1 | 1 | 1 | 1 | 1 | 1 | 0 | | 1 | 7 |
| Luo, 2022 | 1 | 1 | 1 | 1 | 1 | 1 | 0 | | 0 | 6 |
| Masuda, 2016 | 1 | 1 | 1 | 1 | 1 | 1 | 1 | | 1 | 8 |
| Piorkowski, 2008 | 1 | 1 | 1 | 1 | 1 | 1 | 0 | | 1 | 7 |
| Ullah, 2015 | 1 | 1 | 1 | 1 | 1 | 1 | 1 | | 1 | 8 |
| Randomized controlled studies | Randomization | | | | Blinding | | | Withdrawals | | Quality score |
|  | Was the study described as randomized? | | Was the randomization appropriate? | | Was the study described as double blind? | Was the double blinding appropriate? | | Was there a description of withdrawals and dropouts? | |  |
| Matsuo, 2011 | 1 | | 1 | | 0 | 0 | | 1 | | 3 |
| Piorkowski, 2011 | 1 | | 1 | | 0 | 0 | | 1 | | 3 |
| Rajappan, 2009 | 1 | | 1 | | 0 | 0 | | 1 | | 3 |


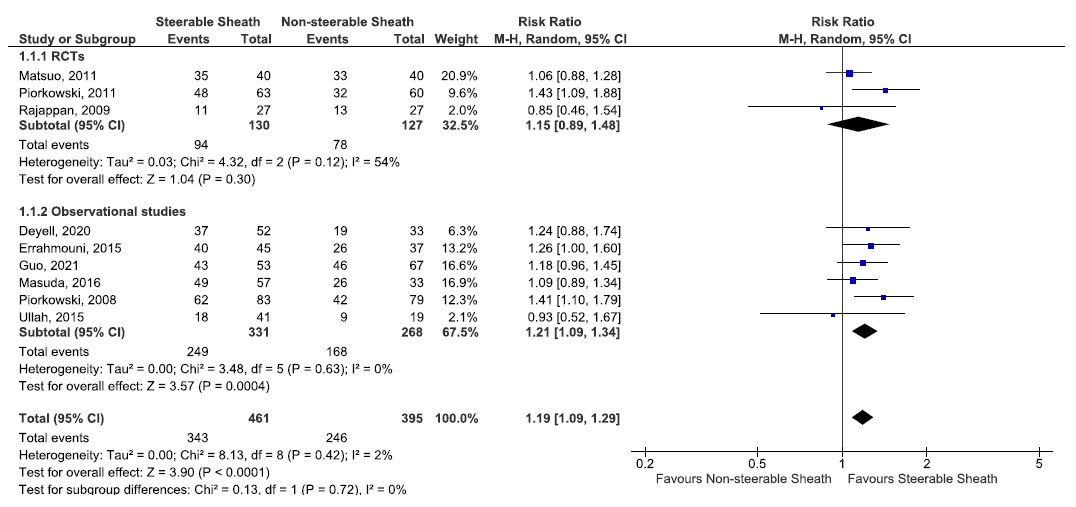


**Supplementary Figure 1:** Subgroup analysis of comparing the steerable and non-steerable sheaths based on the study design (Randomized control trials vs. observational studies)


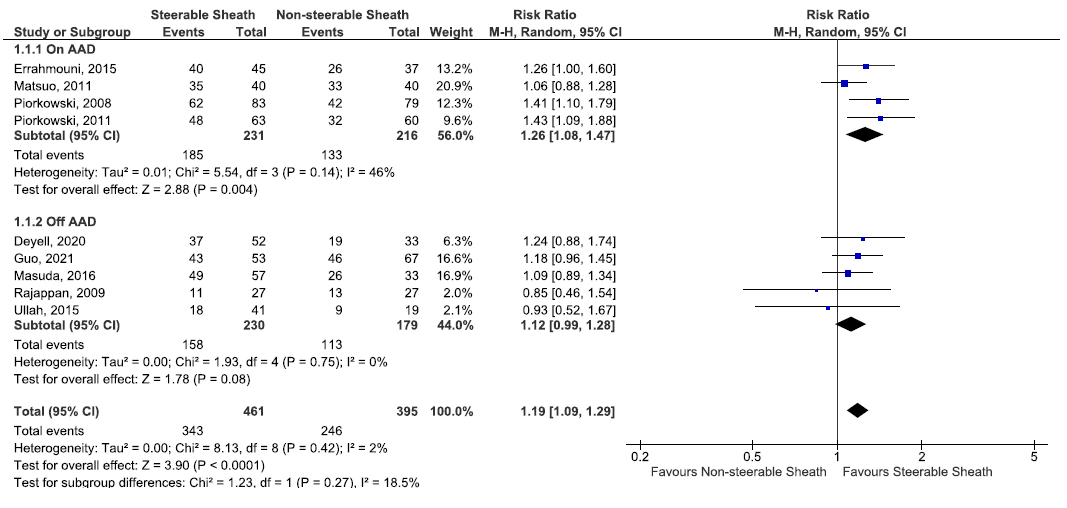


**Supplementary Figure 2:** Subgroup analysis of comparing the steerable and non-steerable sheaths based on whether anti-arrhythmic drugs were allowed or not after the blanking period.


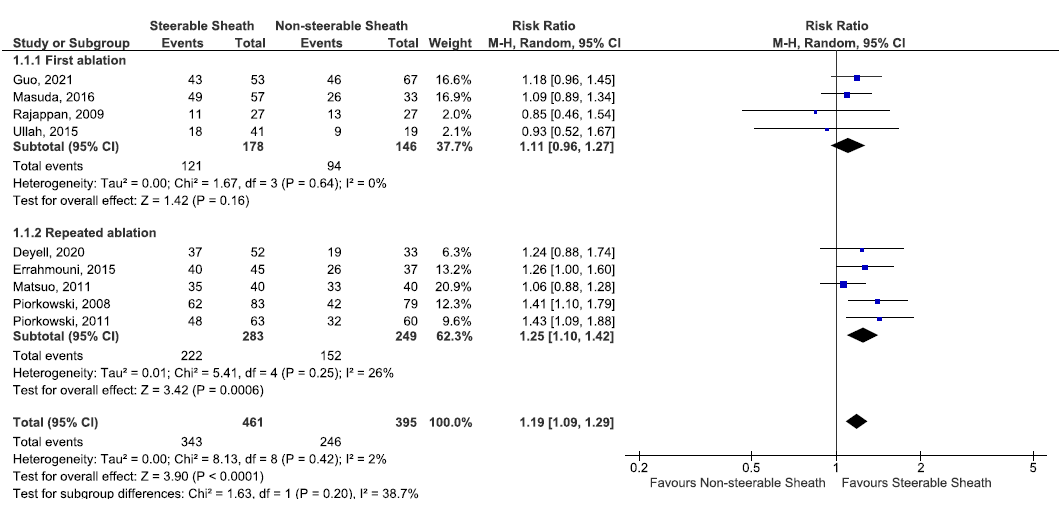


**Supplementary Figure 3:** Subgroup analysis of comparing the steerable and non-steerable sheaths based on ablation status (first time or repeated ablation)

**
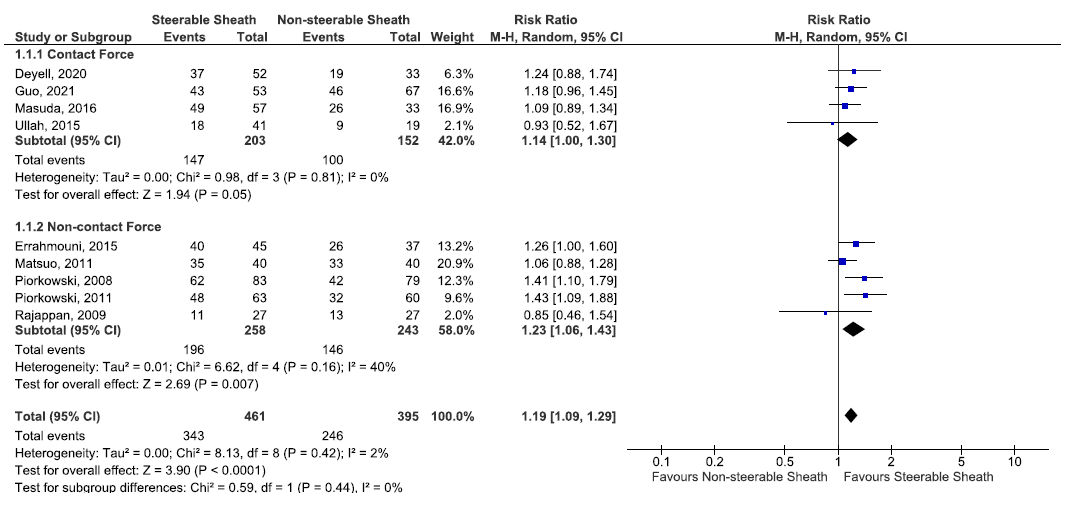
**

**Supplementary Figure 4:** Subgroup analysis of comparing the steerable and non-steerable sheaths based on the use of contact force sensing catheters.

**
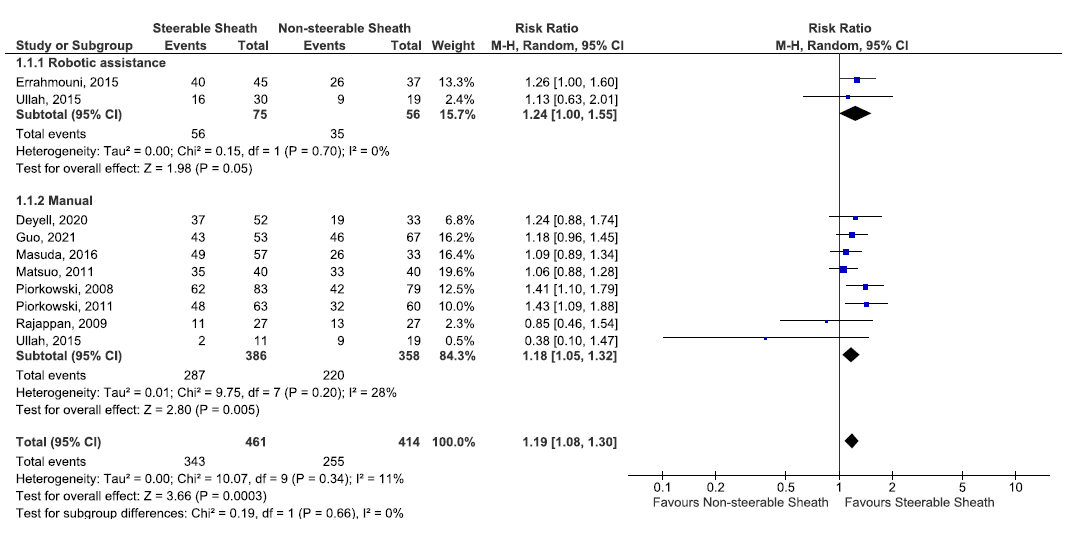
**

**Supplementary Figure 5:** Subgroup analysis of comparing the steerable and non-steerable sheaths based on the steerable sheath’s guidance (robotic assisted vs. manually guided)


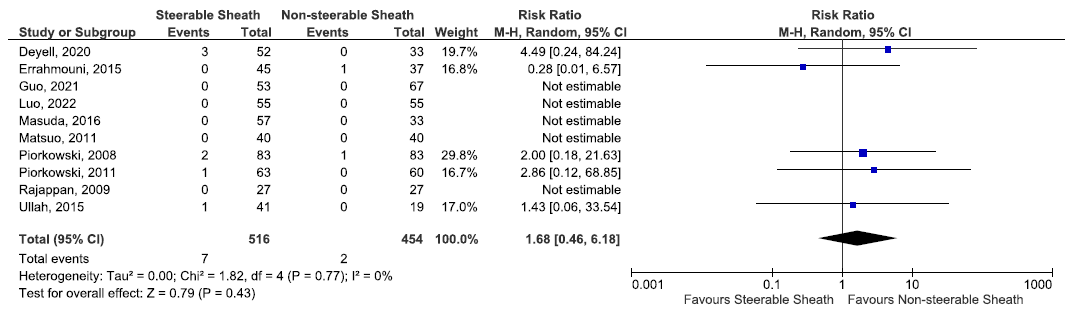


**Supplementary Figure 6:** Forrest blot showing the access-site related complications between the steerable and non-steerable sheath groups.


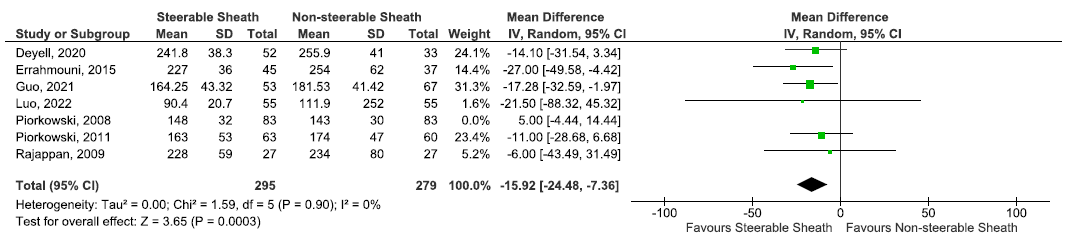


**Supplementary Figure 7:** A sensitivity analysis of the procedure time outcome, which showed no heterogeneity after removing the Poprkowski (2008) study.


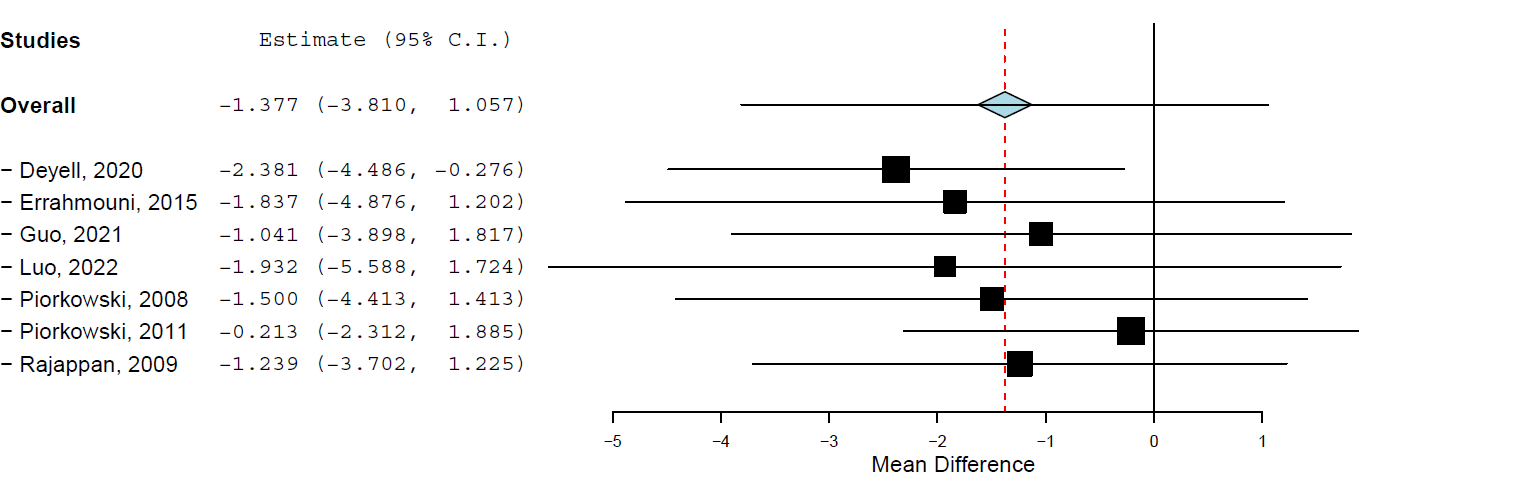


**Supplementary Figure 8:** Leave-one-out sensitivity analysis for the fluoroscopic time.


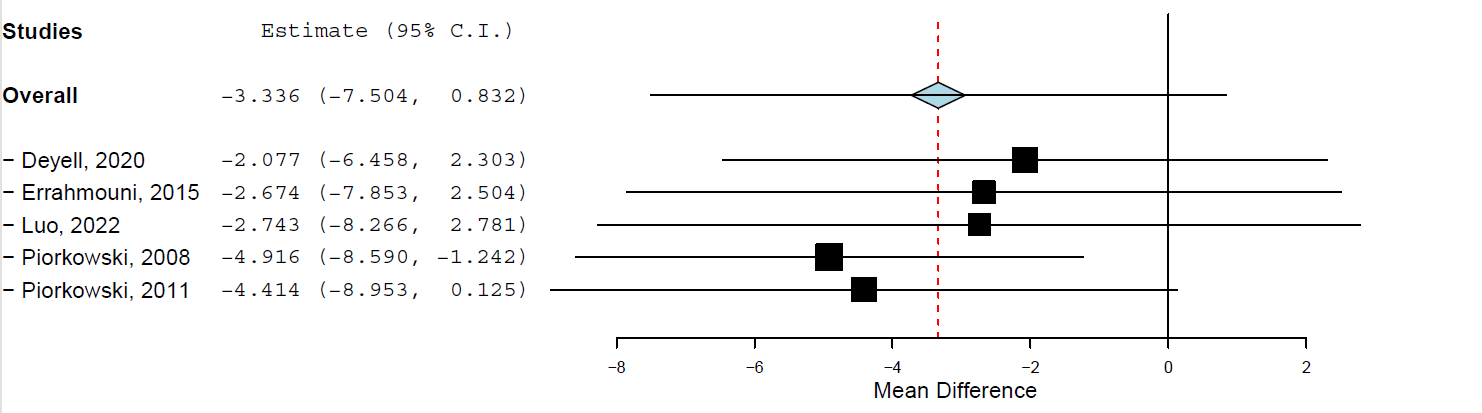


**Supplementary Figure 9:** Leave-one-out sensitivity analysis for the radiofrequency application time.


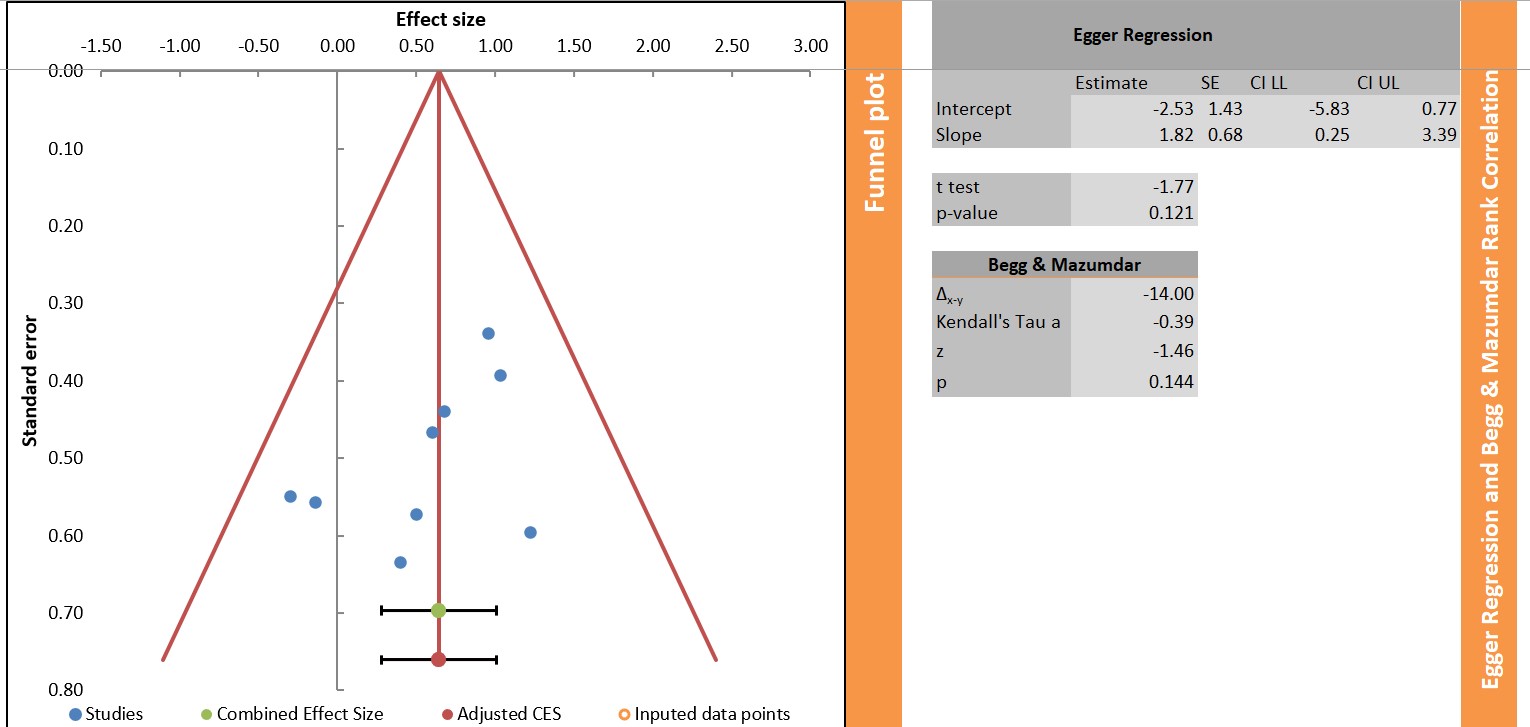


**Supplementary Figure 10:** Funnel plots showing publication bias analysis of studies that assessed the effect of steerable sheath versus non-steerable sheath regarding atrial arrythmia freedom


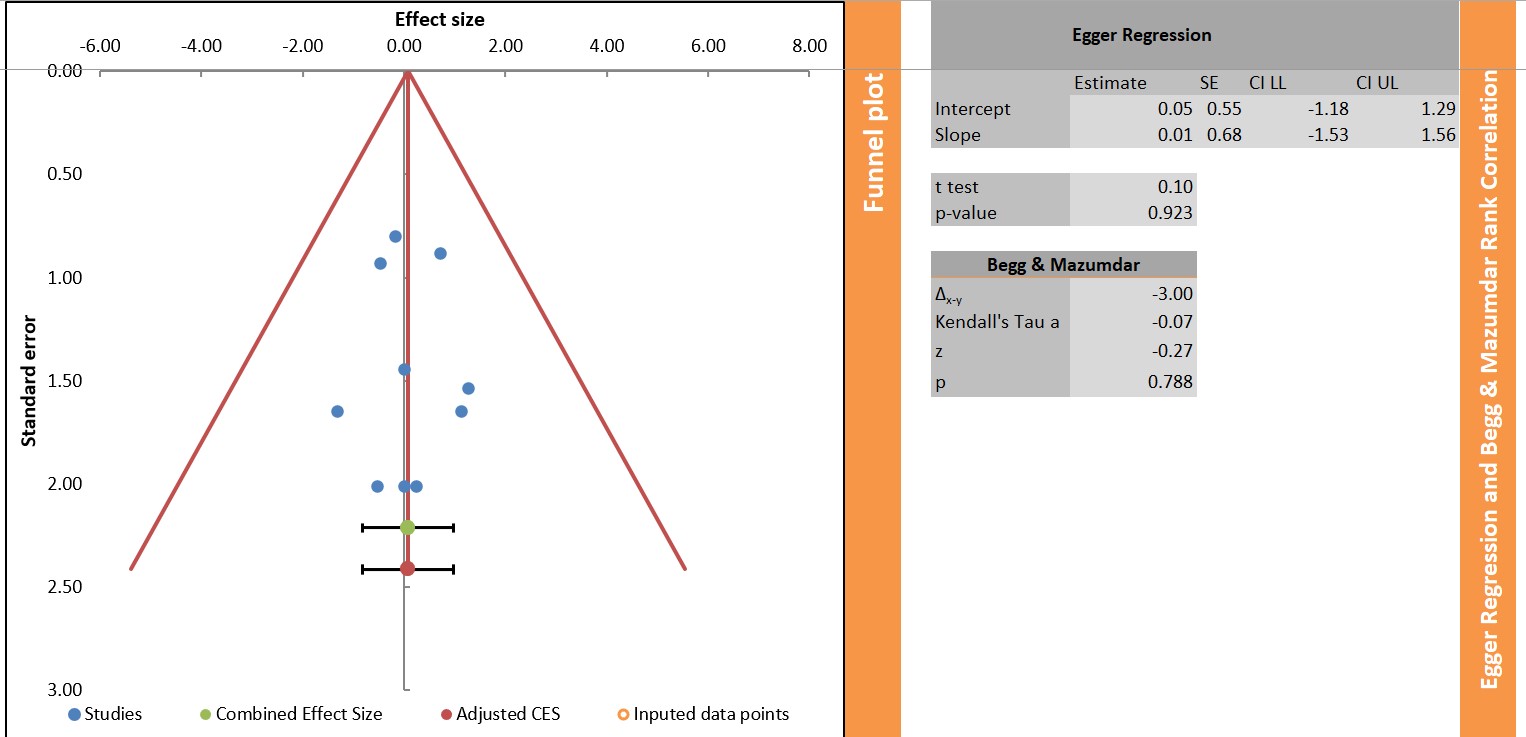


**Supplementary Figure 11:** Funnel plots showing publication bias analysis of studies that assessed the effect of steerable sheath versus non-steerable sheath regarding procedural complications
